# Evaluation of association studies and a systematic review and meta-analysis of VDR polymorphisms in type 2 diabetes mellitus risk

**DOI:** 10.1097/MD.0000000000025934

**Published:** 2021-07-16

**Authors:** Yao Liu, Xin Guo, Shao-Yan Huang, Luan Gong, Jin-Hui Cui, Hu-Wei Shen, Xiang-Hua Ye, Xiao-Feng He

**Affiliations:** a Changzhi Medical College, No. 161, JieFangDong Street; b Department of Endocrinology, Heping Hospital Affiliated to Changzhi Medical College, Shanxi, Changzhi city; c Department of Radiotherapy, First Affiliated Hospital, Zhejiang University School of Medicine, Zhejiang, Hangzhou city; d Institute of Evidence-Based Medicine, Heping Hospital Affiliated to Changzhi Medical College, Shanxi, Changzhi city, PR China.

**Keywords:** meta-analysis, polymorphism, risk, T2DM, VDR

## Abstract

Numerous original studies and 4 published meta-analyses have reported the association between the Vitamin D receptor (*VDR*) BsmI, FokI, ApaI, and TaqI polymorphisms and type 2 diabetes mellitus (T2DM) risk. However, the results were inconsistent. Therefore, an updated meta-analysis was performed to further explore these issues.

To further explore the association between the *VDR* BsmI, FokI, ApaI, and TaqI polymorphisms and T2DM risk.

PubMed, EMBASE, Scopus, and Wanfang databases were searched. The following search strategy were used: (*VDR* OR vitamin D receptor) AND (polymorphism OR variant OR mutation) AND (diabetes OR mellitus OR diabetes mellitus). Pooled crude odds ratios with 95% confidence intervals were applied to evaluate the strength of association in 5 genetic models. Statistical heterogeneity, the test of publication bias, and sensitivity analysis were carried out using the STATA software (Version 12.0). To evaluate the credibility of statistically significant associations, we applied the false-positive report probabilities (FPRP) and Bayesian false discovery probability (BFDP) test.

Overall, the *VDR* BsmI polymorphism was associated with a significantly decreased T2DM risk in Asians; the *VDR* FokI polymorphism was associated with a significantly decreased T2DM risk in Asians, African countries, and Asian countries; the *VDR* ApaI polymorphism was associated with a significantly decreased T2DM risk in Caucasians and North American countries.

On the *VDR* ApaI polymorphism, a significantly increased T2DM risk was found in a mixed population. However, when we further performed a sensitivity analysis, FPRP, and BFDP test, less-credible positive results were identified (all FPRP > 0.2 and BFDP > 0.8) in any significant association.

In summary, this study strongly indicates that all significant associations were less credible positive results, rather than from true associations.

## 1 Introduction

Type 2 diabetes mellitus (T2DM) is a progressive chronic disease that is marked by the inability of tissues such as the liver and skeletal muscles to respond to insulin, it has become a significant global healthcare problem and its reported incidence is increasing at an alarming rate. Based on the recent International Diabetes Federation Diabetes Atlas (9th edition) an estimated 463 million global citizens are suffering from diabetes, costing around 10% of global health spending ($760 billion). Projections based on current trends predict that 700.2 million people will be living with diabetes by 2045; which means that 1 in 11 people will be affected, with an excessive amount of funding required globally to treat diabetes and manage diabetic complications.^[1]^ Therefore, it will be very important to explore the potential pathogenic factors. The pathogenesis of T2DM is complex, many factors such as geography, obesity, diet and exercise, genetic susceptibility, and other possible factors have been discovered, among them, genetic predisposition plays a crucial role in the development of T2DM,^[2]^ although its manifestation is highly dependent on environmental factors.

Vitamin D receptor (*VDR*) was the most extensively reported, which is a member of the nuclear receptor superfamily of transcriptional regulators and located on chromosome 12q13,^[3]^ through binding to vitamin D responsive elements (VDREs and nVDREs), respectively, which is located in the promoter region of target genes to regulates gene transcription positively or negatively.^[4]^ The *VDR* is expressed in many different cell types such as pancreatic b-cells,^[5]^ vascular smooth muscle cells,^[6]^ osteoblasts and chondrocytes,^[7]^ liver, adipose tissue,^[8]^ muscle,^[9]^ dendritic cells, and lymphocytes.^[10]^ Therefore, the *VDR* protein regulates the expression of genes involved in diverse biological functions, and it has also been shown to play a significant role in T2DM.^[11,12]^

Over the past several years, more than 25 *VDR* polymorphism genes have been identified,^[13]^ BsmI, FokI, ApaI, and TaqI are the most studied genes with T2DM, but their relationships are still controversial and uncertain. There also reported several related meta-analyses on the *VDR* BsmI, FokI, ApaI, and TaqI polymorphisms with the risk of T2DM,^[14–17]^ however, their results were also inconsistent. And the literature quality assessments had not been performed or there's no definite number in their studies.^[14–17]^ Moreover, previously published meta-analyses also did not evaluate positive results to identify multiple comparisons. Hence, to further clarify the existing epidemiological evidence and analyze the relationship between *VDR* genetic polymorphisms (BsmI, FokI, ApaI, and TaqI) and T2DM risk, this study systematically reviewed the literature again and conducted an updated meta-analysis. this study included more studies and reliable results than previously published meta-analysis.^[14–17]^

## 2 Materials and methods

### 2.1 Search strategy

We performed the current study according to the guidelines of the PRISMA group.^[18]^ We searched databases including PubMed, EMBASE, Scopus, and the Chinese Wanfang Data Knowledge Service Platform. In addition, we also searched the Catalog of Published Genome-Wide Association Studies (www.genome.gov/gwastudies) of the US National Human Genome Research Institute. The following search strategy were used: (VDR OR vitamin D receptor) AND (polymorphism OR variant OR mutation) AND (diabetes OR mellitus OR diabetes mellitus). The search deadline is September 12, 2020. In addition, the reference lists of previously published meta-analysis^[14–17]^ were also checked.

### 2.2 Selection criteria

The inclusion criteria were as follows: (1) case-control or cohort studies; (2) described the association on the *VDR* BsmI, FokI, ApaI, and TaqI polymorphisms with T2DM risk; and (3) provided sufficient genotype data or the odds ratio (OR) with their 95% confidence intervals (CI) in the selected literature. The exclusion criteria were: (1) duplicated studies or data; (2) studies with no available data; and (3) case reports, reviews, and letters.

### 2.3 Data extraction and quality score assessment

Data were extracted and checked by 2 investigators independently. Disagreement was settled through discussion and consensus. The extracted information was as follows: (1) first author, (2) year of publication, (3) country, (4) geographic region, (5) ethnicity, (6) sample size of cases and controls, (7) source of controls, (8) type of controls, (9) matching, and (10) genotype distributions in cases and controls.

The quality score assessment of selected studies was also independently conducted by 2 authors. Table 1, Supplemental Digital Content lists the scale for quality assessment of molecular association studies of T2DM. The total score was 18 points, studies scoring >11 were high, those scoring <8 were low, and those scoring between 8 and 11 were moderate.

### 2.4 Statistical analysis

The crude ORs with their corresponding 95% CIs were employed to evaluate the strength of association between the *VDR* genetic polymorphisms (BsmI, FokI, ApaI, and TaqI) and T2DM risk. *P* < .05 was considered as statistically significant results. Five genetic models were used: (1) an allele model; (2) an additive model; (3) a dominant model; (4) a recessive model; and (5) an over-dominant model. Heterogeneity among studies applied Chi-square-based *Q* test and *I*^2^ value. There was no obvious heterogeneity among studies if *P* > .10 and/or *I*^2^ ≤ 50%^[19]^ and the ORs were pooled to apply a fixed-effects model.^[20]^ Otherwise, a random-effects model was conducted.^[21]^ Furthermore, a meta-regression analysis was applied to explore sources of heterogeneity. Subgroup analyses were performed by geographic region and ethnicity. We assessed sensitivity analysis by including high-quality and Hardy–Weinberg equilibrium (HWE) in control studies. HWE was examined using Chi-square goodness-of-fit test and it was regarded as HWE in controls if *P* > .05. The publication bias was estimated using the Begg funnel plot and Egger test.^[22]^ A nonparametric “trim and fill” method^[23]^ will be employed to add missing studies if an obvious publication bias was found. Finally, the false-positive report probabilities (FPRP)^[24]^ and the Bayesian False Discovery Probability (BFDP) test^[25]^ were applied to assess the credibility of statistically significant associations. We preset a noteworthy value (FPRP < 0.2 and BFDP < 0.8) and set a prior probability of 0.01 to detect risk.^[24,23]^ All statistical analyses were conducted using Stata 12.0 software (STATA Corporation, CollegeStation, TX).

## 3 Results

### 3.1 Search results and study characteristics

**Figure 1:**
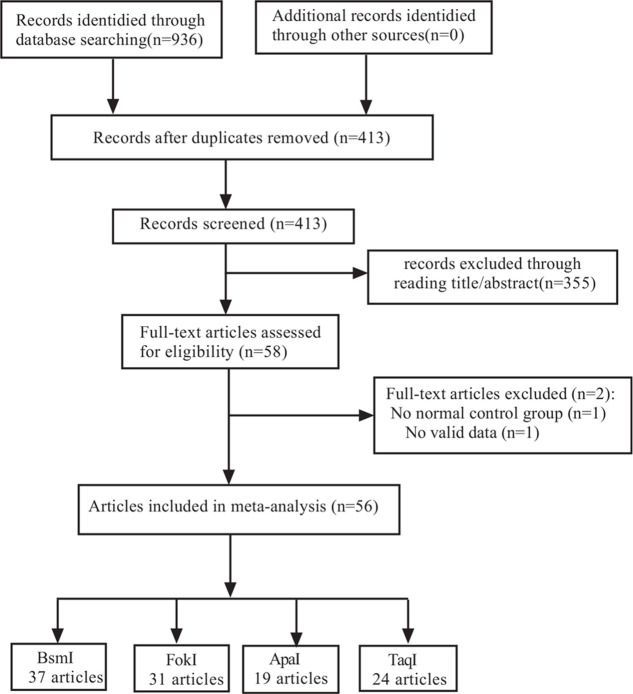
Flow diagram for identifying and including studies in the current meta-analysis.

Figure 1 shows a more detailed search process. These searches returned 936 records, of which 355 were excluded as irrelevant based on the reading of the title and abstract. The remaining 58 articles were read in full by the 2 authors independently. Two studies were excluded because of no normal control group and valid data. As a result, 56 studies met these requirements and were included in this study. The current and previously published meta-analyses involving studies were shown in Table 2, Supplemental Digital Content. 56 studies met our requirements,^[26,27,28–34,19,35–80]^ of which 37 studies reported the *VDR* BsmI (5586 cases and 6484 controls), 31 studies examined the *VDR* FokI (6525 cases and 7464 controls), 19 studies investigated the *VDR* ApaI (2593 cases and 3557 controls), and 24 studies explored the *VDR* TaqI (3221 cases and 4027 controls) with T2DM risk, as shown in Figure 1 and Table 4, Supplemental Digital Content. Among these studies, 25, 22, 6, and 4 of the studies were conducted to analyze Asians, Caucasians, Indians, and mixed populations, respectively. Finally, there were 12 high-quality studies, 19 medium-quality studies, and 6 low-quality studies on the *VDR* BsmI, 13 high-quality studies, 15 medium-quality studies, and 2 low-quality studies on the *VDR* FokI, 10 high-quality studies, 9 medium-quality studies on the *VDR* ApaI, and 13 high-quality studies and 11 medium-quality studies on the *VDR* TaqI. The detailed characteristics and scoring of each study are displayed in Table 4, Supplemental Digital Content. The genotype frequencies of *VDR* BsmI, FokI, ApaI, and TaqI polymorphisms with T2DM risk and HWE test results were shown in Table 4, Supplemental Digital Content.

### 3.2 Quantitative synthesis

Table 1 shows the results on the association between the *VDR* BsmI and T2DM risk. No significant association was observed in the overall analysis. Subgroups were conducted by ethnicity and geographic region, the *VDR* BsmI was associated with a significantly decreased T2DM risk in Asians (BB vs (Bb + bb): OR = 0.77, 95% CI = 0.60–0.99). Unfortunately, after FPRP and BFDP correction, less-credible results were found in Asians, as shown in Table 5.

**Table 1:** Pooled results on the association between the VDR BsmI polymorphism and T2DM risk.

		Bb vs bb		BB vs bb		(BB + Bb) vs bb		BB vs (Bb + bb)		B vs b	
Variable	n (Cases/Controls)	OR (95% CI)	*P*_h_/*I*^2^(%)	OR (95% CI)	*P*_h_/*I*^2^(%)	OR (95% CI)	*P*_h_/*I*^2^(%)	OR (95% CI)	*P*_h_/*I*^2^(%)	OR (95% CI)	*P*_h_/*I*^2^(%)
Overall	37 (5586/6484)	1.07 (0.98, 1.17)	0.000/72.8%	1.69 (0.94, 1.19)	0.000/61.1%	–	0.000/77.2%	–	0.000/81.1%	–	0.000/80.2%
Ethnicity											
Asian	15 (2276/2430)	–	0.000/77.4%	0.93 (0.75, 1.15)	0.029/46.3%	–	0.000/83.6%	**0.77 (0.60, 0.99)**	0.058/40.5%	–	0.000/85.3%
Caucasian	14 (2467/3172)	1.04 (0.96, 1.13)	0.014/51.2%	1.00 (0.93, 1.09)	0.220 /22.1%	1.02 (0.97, 1.08)	0.008/54%	0.93 (0.84, 1.02)	0.520/0%	1.00 (0.96, 1.04)	0.043/43.2%
Indian	6 (680/670)	–	0.000/90%	–	0.000/86.8%	–	0.000/89.7%	–	0.000/85.1%	–	0.000/90.6%
Mixed	2 (163/212)	1.00 (0.75, 1.35)	0.449/0.0%	1.11 (0.73, 1.70)	0.703/0%	1.03 (0.83, 1.27)	0.748/0%	1.10 (0.71, 1.71)	0.461/0%	1.05 (0.85, 1.29)	0.820/0%
Geographic region											
Africa	3 (204/260)	1.04 (0.86, 1.25)	0.267/24.3%	0.95 (0.80, 1.12)	0.342/6.9%	1.00 (0.91, 1.11)	0.233/31.3%	0.90 (0.72, 1.12)	0.625/0.0%	0.96 (0.87, 1.07)	0.306/15.5%
Asia	26 (4109/4043)	–	0/80.8%	1.16 (0.95, 1.42)	0/72.8%	–	0/84.4%	–	0/77.1%	–	0/85.7%
Europe	5 (792/644)	0.99 (0.89, 1.10)	0.333/12.7%	0.97 (0.80, 1.19)	0.307/16.8%	0.98 (0.91, 1.06)	0.230/28.7%	0.96 (0.77, 1.20)	0.789/0%	0.98 (0.89, 1.07)	0.330/13.2%
South America	2 (239/234)	0.95 (0.77, 1.18)	0.953/0.0%	1.08 (0.81, 1.43)	0.584/0.0%	1.00 (0.87, 1.15)	0.806/0.0%	1.15 (0.82, 1.61)	0.811/0.0%	1.04 (0.89, 1.21)	0.778/0.0%
North America	1 (242/1303)	0.98 (0.86, 1.13)	–	1.02 (0.80, 1.31)	–	1.00 (0.90, 1.10)	–	1.04 (0.79, 1.37)	–	1.01 (0.90, 1.13)	–
Sensitivity analysis											
Overall	12 (1883/2111)	1.10 (0.93, 1.30)	0.001/65.1%	1.03 (0.88, 1.21)	0.412/3.3%	1.08 (0.94, 1.24)	0/68.4%	1.01 (0.83, 1.23)	0.528/0.0%	1.09 (0.93, 1.27)	0.001/66.7%
Ethnicity											
Asian	7 (1234/1529)	1.01 (0.90, 1.13)	0.000/75.8%	1.84 (0.83, 4.07)	0.332/12.7%	–	0.000/77.1%	1.70 (0.76, 3.82)	0.375/6.9%	–	0.000/77%
Caucasian	3 (509/402)	0.95 (0.84, 1.07)	0.929/0.0%	0.92 (0.77, 1.09)	0.691/0.0%	0.96 (0.88, 1.04)	0.781/0.0%	0.93 (0.75, 1.16)	0.681/0.0%	0.95 (0.86, 1.04)	0.699/0.0%
Indian	2 (140/180)	1.21 (0.97, 1.49)	0.217/34.4%	1.29 (0.81, 2.03)	0.659/0.0%	1.16 (0.98, 1.37)	0.390/0.0%	1.11 (0.67, 1.84)	0.304/5.2%	1.05 (0.95, 1.39)	0.915/0.0%
Mixed	–	–	–	–	–	–	–	–	–	–	–
Geographic region											
Africa	–	–	–	–	–	–	–	–	–	–	–
Asia	10 (1474/1809)	1.18 (0.93, 1.49)	0.001/68%	1.14 (0.93, 1.40)	0.209/25.5%	1.16 (0.94, 1.43)	0.000/72.9%	1.05 (0.82, 1.34)	0.388/5.7%	1.15 (0.93, 1.44)	0.000/71.4%
Europe	1 (308/240)	0.93 (0.79, 1.10)	–	0.82 (0.54, 1.24)	–	0.93 (0.81, 1.07)	–	0.85 (0.54, 1.33)	–	0.92 (0.78, 1.08)	–
South America	1 (101/62)	0.96 (0.78, 1.18)	–	1.00 (0.74, 1.36)	–	0.98 (0.86, 1.12)	–	1.10 (0.71, 1.73)	–	1.02 (0.85, 1.22)	–

CI = confidence interval, OR = odds ratio, T2DM = type 2 diabetes mellitus, VDR = vitamin D receptor. The positive results of VDR polymorphisms and type 2 diabetes mellitus risk.

Table 2 summarizes the results on the association between the *VDR* FokI and T2DM risk. Overall, a significantly decreased T2DM risk was observed in overall analysis (FF vs ff: OR = 0.90, 95% CI = 0.84–0.96; (FF + Ff) vs ff: OR = 0.98, 95% CI = 0.96–1.00), Asians (FF vs ff: OR = 0.87, 95% CI = 0.82–0.93; (FF + Ff) vs ff: OR = 0.97, 95% CI = 0.94–0.99; FF vs (Ff + ff): OR = 0.79, 95% CI = 0.69–0.90; F vs f: OR = 0.93, 95% CI = 0.90–0.96), African countries (FF vs ff: OR = 0.77, 95% CI = 0.62–0.96), and Asian countries (FF vs ff: OR = 0.91, 95% CI = 0.84–0.98; (FF + Ff) vs ff: OR = 0.97, 95% CI = 0.95–1.00; FF vs (Ff + ff): OR = 0.84, 95% CI = 0.75–0.95, F vs f: OR = 0.94, 95% CI = 0.89–0.99). After FPRP and BFDP correction, associations remained significant in the overall population, Asians, and African countries, as shown in Table 5.

**Table 2:** Pooled results on the association between the VDR *FokI* polymorphism and T2DM risk.

		Ff vs ff		FF vs ff		(FF + Ff) vs ff		FF vs (Ff + ff)		F vs f	
Variable	n (cases/controls)	OR (95% CI)	*P*_h_/*I*^2^ (%)	OR (95% CI)	*P*_h_/*I*^2^(%)	OR (95% CI)	*P*_h_/*I*^2^(%)	OR (95% CI)	*P*_h_/*I*^2^(%)	OR (95% CI)	*P*_h_/*I*^2^(%)
Overall	31 (6525/7464)	0.99 (0.96, 1.02)	0.127/22.9%	**0.90 (0.84, 0.96)**	0.001/51.6%	**0.98 (0.96, 1.00)**	0.004/45.4%	0.83 (0.75, 0.92)	0.000/62.4%	0.93 (0.89, 0.97)	0.000/67.6%
Ethnicity											
Asian	15 (2925/4263)	0.98 (0.94, 1.02)	0.694/0%	**0.87 (0.82, 0.93)**	0.095/34.2%	**0.97 (0.94, 0.99)**	0.497/0%	**0.79 (0.69, 0.90)**	0.008/53.3%	**0.93 (0.90, 0.96)**	0.027/45.9%
Caucasian	13 (3202/2709)	0.98 (0.93, 1.03)	0.131/31.5%	0.94 (0.85, 1.04)	0.002/60.8%	0.97 (0.92, 1.02)	0.002/60.6%	0.90 (0.77, 1.06)	0.000/67.2%	–	0.000/77.9%
Indian	1 (100/160)	1.23 (0.99, 1.54)	–	4.05 (0.38, 44.34)	–	0.19 (0.07, 0.49)	–	7.62 (0.71, 82.02)	–	0.28 (0.13, 0.62)	–
Mixed	2 (298/332)	1.12 (0.91, 1.38)	0.078/67.9%	–	0.022/80.9%	–	0.022/81%	0.59 (0.35, 1.02)	0.085/66.3%	–	0.012/84%
Geographic region											
Africa	4 (643/562)	0.99 (0.88, 1.13)	0.179/38.7%	**0.77 (0.62, 0.96)**	0.084/54.9%	0.90 (0.78, 1.03)	0.009/74.2%	0.67 (0.56, 0.81)	0.105/51.1%	–	0.000/84.5%
Asia	20 (4094/5226)	0.98 (0.95, 1.02)	0.233/17.8%	**0.91 (0.84, 0.98)**	0.005/50.5%	**0.97 (0.95, 1.00)**	0.047/37.5%	**0.84 (0.75, 0.95)**	0.001/56.9%	**0.94 (0.89, 0.99)**	0.000/60.7%
Europe	3 (1307/1182)	1.00 (0.93, 1.08)	0.234/31.2%	1.03 (0.89, 1.19)	0.674/0%	1.01 (0.95, 1.07)	0.471/0%	1.07 (0.81, 1.42)	0.085/59.4%	1.01 (0.95, 1.08)	0.600/0.0%
South America	4 (481/494)	1.06 (0.96, 1.18)	0.109/50.5%	0.88 (0.62, 1.24)	0.028/67.1%	1.01 (0.90, 1.14)	0.057/60.1%	0.74 (0.48, 1.16)	0.021/69.0%	0.93 (0.79, 1.11)	0.024/68.2%
Sensitivity analysis											
Overall	12 (2357/3959)	1.00 (0.96, 1.04)	0.349/9.8%	**0.93 (0.87, 1.00)**	0.288/15.9%	0.99 (0.96, 1.02)	0.201/24.7%	**0.89 (0.81, 0.97)**	0.229/21.8%	0.96 (0.92, 1.00)	0.098/36.6%
Ethnicity											
Asian	6 (1559/3154)	0.98 (0.93, 1.03)	0.672/0.0%	**0.92 (0.83, 1.00)**	0.093/47.0%	0.98 (0.94, 1.01)	0.344/11.1%	0.91 (0.81, 1.02)	0.117/43.3%	0.96 (0.92, 1.01)	0.114/43.7%
Caucasian	5 (660/633)	0.99 (0.91, 1.09)	0.626/0%	0.94 (0.85, 1.04)	0.495/0%	0.98 (0.93, 1.03)	0.409/0%	**0.85 (0.74, 0.98)**	0.386/3.6%	**0.94 (0.88, 1.00)**	0.173/37.3%
Indian	–	–	–	–	–	–	–	–	–	–	–
Mixed	1 (138/172)	**1.25 (1.05, 1.48)**	–	1.11 (0.76, 1.62)	–	**1.15 (1.01, 1.31)**	–	0.79 (0.50, 1.25)	–	1.06 (0.90, 1.26)	–
Geographic region											
Africa	1 (87/150)	0.94 (0.77, 1.14)	–	**0.85 (0.71, 1.00)**	–	0.93 (0.85, 1.02)	–	0.69 (0.53, 0.91)	–	**0.84 (0.74, 0.95)**	–
Asia	7 (1641/3236)	0.98 (0.94, 1.03)	0.785/0%	**0.92 (0.84, 1.00)**	0.139/38%	0.98 (0.94, 1.01)	0.465/0%	0.91 (0.82, 1.01)	0.183/32.1%	**0.96 (0.92, 1.00)**	0.178/32.7%
Europe	1 (308/239)	1.05 (0.92, 1.20)	–	0.96 (0.78, 1.17)	–	1.01 (0.93, 1.11)	–	0.86 (0.66, 1.11)	–	0.98 (0.87, 1.08)	–
South America	3 (321/334)	1.06 (0.85, 1.33)	0.076/61.3%	1.04 (0.84, 1.29)	0.627/0%	1.05 (0.96, 1.15)	0.101/56.4%	0.92 (0.70, 1.21)	0.496/0%	1.02 (0.91, 1.14)	0.314/13.6%

CI = confidence interval, OR = odds ratio, T2DM = type 2 diabetes mellitus, VDR = vitamin D receptor. The positive results of VDR polymorphisms and type 2 diabetes mellitus risk.

The results of the association on the *VDR* ApaI with T2DM risk are shown in Table 3. No significantly decreased T2DM risk was found in the overall analysis. Then, subgroup analyses result observed a significantly decreased T2DM risk in Caucasian (Aa vs aa: OR = 0.94, 95% CI = 0.89–0.99; (Aa + AA) vs aa: OR = 0.96, 95% CI = 0.93–1.00) and North American countries (Aa vs aa: OR = 0.90, 95% CI = 0.81–1.00). In addition, a significantly increased T2DM risk was observed in mixed populations (AA vs (Aa + aa): OR = 1.52, 95% CI = 1.04–2.22). After FPRP and BFDP correction, less-credible results were found in Caucasian, North American countries, and mixed populations, as also shown in Table 5.

**Table 3:** Pooled results on the association between the VDR ApaI polymorphism and T2DM risk.

		Aa vs aa		AA vs aa		(Aa + AA) vs aa		AA vs (Aa + aa)		A vs a	
Variable	n (Cases/Controls)	OR (95% CI)	*P*_h_/*I*^2^(%)	OR (95% CI)	*P*_h_/*I*^2^(%)	OR (95% CI)	*P*_h_/*I*^2^(%)	OR (95% CI)	*P*_h_/*I*^2^(%)	OR (95% CI)	*P*_h_/*I*^2^(%)
Overall	19 (2595/3557)	0.97 (0.93, 1.01)	0.522/0%	0.96 (0.89, 1.03)	0.209/20.1%	0.98 (0.95, 1.01)	0.408/3.9%	1.03 (0.94, 1.13)	0.259/16%	0.99 (0.95, 1.03)	0.285/13.8%
Ethnicity											
Asian	7 (680/702)	1.03 (0.93, 1.15)	0.482/0.0%	0.95 (0.75, 1.21)	0.178/32.8%	1.01 (0.92, 1.10)	0.216/27.8%	0.92 (0.70, 1.20)	0.547/0.0%	0.99 (0.90, 1.09)	0.145/37.1%
Caucasian	10 (1701/2630)	**0.94 (0.89, 0.99)**	0.511/0.0%	0.94 (0.86, 1.01)	0.291/16.5%	**0.96 (0.93, 1.00)**	0.588/0.0%	1.01 (0.91, 1.12)	0.220/24.3%	0.98 (0.93, 1.02)	0.576/0.0%
Indian	1 (89/100)	1.04 (0.80, 1.35)	–	1.13 (0.73, 1.75)	–	1.04 (0.87, 1.25)	–	1.12 (0.67, 1.88)	–	1.06 (0.86, 1.32)	–
Mixed	1 (125/125)	0.99 (0.86, 1.14)	–	1.17 (0.91, 1.50)	–	1.02 (0.93, 1.12)	–	**1.52 (1.04, 2.22)**	–	1.13 (0.98, 1.30)	–
Geographic region											
Asia	13 (1489/1643)	–	0.000/77.9%	0.96 (0.86, 1.06)	0.245/19.7%	0.99 (0.95, 1.04)	0.329/11.6%	0.99 (0.87, 1.13)	0.417/3.0%	0.99 (0.94, 1.05)	0.435/1.1%
Europe	3 (638/4241)	0.95 (0.86, 1.04)	0.941/0%	0.99 (0.84, 1.17)	0.426/0.0%	0.97 (0.91, 1.04)	0.755/0.0%	1.09 (0.88, 1.35)	0.291/18.9%	0.99 (0.94, 1.05)	0.358/2.7%
South America	1 (121/62)	0.97 (0.63, 1.49)	–	2.35 (0.53, 10.54)	–	1.05 (0.71, 1.55)	–	2.46 (0.54, 11.19)	–	1.16 (0.76, 1.76)	–
North America	2 (367/1428)	**0.90 (0.81, 1.00)**	0.135/55.3%	–	0.038/76.7%	0.96 (0.86, 1.08)	0.061/71.5%	–	0.023/80.6%	–	0.017/82.5%
Sensitivity analysis											
Overall	10 (1222/1207)	1.02 (0.95, 1.10)	0.661/0.0%	0.99 (0.88, 1.13)	0.185/28.2%	1.01 (0.96, 1.07)	0.439/0.0%	0.97 (0.83, 1.14)	0.464/0.0%	1.00 (0.94, 1.07)	0.346/10.5%
Ethnicity											
Asian	4 (452/436)	1.07 (0.94, 1.21)	0.345/9.6%	0.95 (0.58, 1.54)	0.080/55.7%	1.04 (0.94, 1.15)	0.156/42.5%	0.95 (0.69, 1.30)	0.250/26.9%	1.01 (0.85, 1.22)	0.086/54.6%
Caucasian	5 (681/671)	0.99 (0.91, 1.08)	0.616/0.0%	0.97 (0.83, 1.13)	0.237/27.7%	0.99 (0.93, 1.06)	0.557/0.0%	0.96 (0.79, 1.16)	0.362/7.8%	0.98 (0.91, 1.06)	0.592/0.0%
Indian	1 (89/100)	1.04 (0.80, 1.35)	–	1.13 (0.73, 1.75)	–	1.04 (0.87, 1.25)	–	1.12 (0.67, 1.88)	–	1.06 (0.86, 1.32)	–
Mixed	–	–	–	–	–	–	–	–	–	–	–
Geographic region											
Asia	8 (813/905)	1.06 (0.97, 1.15)	0.774/0.0%	1.02 (0.88, 1.19)	0.208/27.6%	1.03 (0.97, 1.10)	0.529/0.0%	0.97 (0.80, 1.17)	0.411/2.5%	1.02 (0.95, 1.10)	0.317/14.4%
Europe	1 (308/240)	0.94 (0.82, 1.07)	–	0.89 (0.69, 1.13)	–	0.95 (0.86, 1.05)	–	0.92 (0.68, 1.24)	–	0.94 (0.84, 1.06)	–
South America	1 (101/62)	0.97 (0.63, 1.49)	–	2.35 (0.53, 10.54)	–	1.05 (0.71, 1.55)	–	2.46 (0.54, 11.19)	–	1.16 (0.76, 1.76)	–

CI = confidence interval, OR = odds ratio, T2DM = type 2 diabetes mellitus, VDR = vitamin D receptor. The positive results of VDR polymorphisms and type 2 diabetes mellitus risk.

As lists in Table 4, there was no significant association in overall and subgroup analyses on the *VDR* TaqI polymorphism with T2DM risk.

**Table 4:** Pooled results on the association between the VDR TaqI polymorphism and T2DM risk.

		Tt vs tt		TT vs tt		(Tt + TT) vs tt		TT vs (Tt + tt)		T vs t	
Variable	n (Cases/Controls)	OR (95% CI)	*P*_h_/*I*^2^(%)	OR (95% CI)	*P*_h_/*I*^2^(%)	OR (95% CI)	*P*_h_/*I*^2^(%)	OR (95% CI)	*P*_h_/*I*^2^(%)	OR (95% CI)	*P*_h_/*I*^2^(%)
Overall	24 (3221/4027)	1.00 (0.94, 1.07)	0.001/54.1%	0.98 (0.93, 1.03)	0.000/72.0%	1.00 (0.97, 1.02)	0.001/53.4%	0.96 (0.88, 1.06)	0.000/65.5%	0.99 (0.95, 1.03)	0.000/63.8%
Ethnicity											
Asian	5 (621/612)	–	0.001/77.6%	0.99 (0.97, 1.01)	0.134/46.3%	–	0.000/86.7%	1.03 (0.98, 1.08)	0.785/0.0%	1.00 (0.97, 1.03)	0.156/39.8%
Caucasian	13 (2105/2870)	1.03 (0.94, 1.13)	0.000/65.8%	0.93 (0.83, 1.05)	0.000/68.2%	1.00 (0.95, 1.06)	0.000/65.7%	0.88 (0.75, 1.05)	0.000/69.2%	0.97 (0.90, 1.04)	0.000/68.9%
Indian	5 (370/420)	1.00 (0.87, 1.14)	0.703/0.0%	1.00 (0.75, 1.33)	0.063/55.1%	0.99 (0.91, 1.07)	0.323/14.3%	1.07 (0.65, 1.77)	0.003/74.8%	1.03 (0.83, 1.27)	0.003/74.8%
Mixed	1 (125/125)	0.90 (0.76, 1.07)	–	0.94 (0.71, 1.25)	–	0.94 (0.84, 1.06)	–	1.12 (0.76, 1.65)	–	0.99 (0.84, 1.15)	–
Geographic region											
Africa	1 (50/50)	1.05 (0.78, 1.40)	–	0.95 (0.70, 1.29)	–	1.00 (0.85, 1.17)	–	0.83 (0.52, 1.31)	–	0.94 (0.76, 1.16)	–
Asia	16 (1983/1964)	0.98 (0.87, 1.10)	0.000/66.6%	–	0.000/93.7%	0.98 (0.94, 1.02)	0.000/71.9%	–	0.000/80.1%	–	0.000/78.8%
Europe	3 (638/423)	1.02 (0.94, 1.11)	0.869/0.0%	1.06 (0.96, 1.17)	0.704/0.0%	1.02 (0.97, 1.07)	0.786/0.0%	1.10 (0.95, 1.29)	0.678/0.0%	1.05 (0.98, 1.12)	0.627/0.0%
South America	2 (183/162)	1.09 (0.95, 1.26)	0.619/0.0%	1.11 (0.95, 1.29)	0.597/0.0%	1.05 (0.98, 1.14)	0.992/0.0%	1.06 (0.82, 1.35)	0.203/38.3%	1.06 (0.82, 1.35)	0.343/0.0%
North America	2 (367/1428)	0.97 (0.89, 1.06)	0.317/0.0%	0.99 (0.88, 1.10)	0.713/0.0%	0.99 (0.93, 1.04)	0.395/0.0%	1.02 (0.87, 1.19)	0.595/0.0%	0.99 (0.93, 1.07)	0.894/0.0%
Sensitivity analysis											
Overall	12 (1421/1420)	0.94 (0.84, 1.05)	0.001/64.4%	–	0.000/81.5%	–	0.000/83.8%	1.00 (0.87, 1.15)	0.000/71.3%	–	0.000/81.6%
Ethnicity											
Asian	3 (489/429)	–	0.000/91.9%	1.00 (0.97, 1.02)	0.095/64.1%	–	0.000/94.6%	1.02 (0.97, 1.08)	0.741/0.0%	1.00 (0.93, 1.07)	0.034/70.4%
Caucasian	5 (663/671)	–	0.001/79.8%	–	0.000/86.9%	–	0.000/87.0%	–	0.000/80.1%	–	0.000/89.3%
Indian	4 (269/320)	1.01 (0.86, 1.18)	0.560/0.0%	1.09 (0.67, 1.77)	0.051/61.3%	1.00 (0.90, 1.11)	0.267/24.1%	–	0.002/79.6%	–	0.003/78.6%
Mixed	–	–	–	–	–	–	–	–	–	–	–
Geographic region											
Africa	–	–	–	–	–	–	–	–	–	–	–
Asia	9 (930/1018)	0.86 (0.72, 1.03)	0.000/72.4%	–	0.000/95.5%	–	0.000/95.3%	–	0.000/79.1%	–	0.000/88.4%
Europe	1 (308/240)	1.04 (0.93, 1.16)	–	1.10 (0.97, 1.24)	–	1.04 (0.98, 1.10)	–	1.10 (0.97, 1.24)	–	1.08 (0.99, 1.18)	–
South America	2 (183/162)	1.09 (0.95, 1.26)	0.619/0.0%	1.11 (0.95, 1.29)	0.597/0.0%	1.05 (0.98, 1.14)	0.992/0.0%	1.06 (0.82, 1.35)	0.203/38.3%	1.06 (0.82, 1.35)	0.343/0.0%

CI = confidence interval, OR = odds ratio, T2DM = type 2 diabetes mellitus, VDR = vitamin D receptor. The positive results of VDR polymorphisms and type 2 diabetes mellitus risk.

### 3.3 Heterogeneity and sensitivity analyses

Heterogeneity was shown in Tables 1–4. Some potential factors were considered as sources of heterogeneity, such as geographic region, ethnicity, sample size, quality score, and HWE. Then, we applied a meta-regression analysis to investigate sources of heterogeneity. No covariate was identified as a potential source of heterogeneity among studies for the *VDR* BsmI and ApaI. However, we found that ethnicity (FF vs (Ff + ff): *P* = .004; F vs f: *P* < .001), sample size (F vs f: *P* = .016), quality score (FF vs (Ff + ff): *P* = .006; F vs f: *P* = .001), and HWE (FF vs (Ff + ff): *P* = .018; F vs f: *P* = .002) were the source of heterogeneity in the overall analysis for the *VDR* FokI polymorphism. Concerning the *VDR* TaqI polymorphism, the quality of selected studies was the source of heterogeneity in the overall population (Tt vs tt: *P* = .033).

Sensitivity analyses were estimated by applying 2 methods in this meta-analysis. First, results did not change removing a single study each time. Second, when we excluded studies of low quality and Hardy–Weinberg disequilibrium (HWD) in controls, no significantly decreased or increased T2DM risk was observed for the *VDR* BsmI, ApaI, and TaqI polymorphisms, as also shown in Tables 1, 3 and 4.

A significant association was observed in the overall analysis (FF vs ff: OR = 0.93, 95% CI = 0.87–1.00; FF vs (Ff + ff)): OR = 0.89, 95% CI = 0.81–0.97), Asians (FF vs ff: OR = 0.92, 95% CI = 0.83–1.00; FF vs (Ff + ff): OR = 0.85, 95% CI = 0.74–0.98; F vs f: OR = 0.94, 95% CI = 0.88–1.00), African countries (FF vs ff: OR = 0.85, 95% CI = 0.71–1.00; F vs f: OR = 0.84, 95% CI = 0.74–0.95), Asian countries (FF vs ff: OR = 0.92, 95% CI = 0.84–1.00; F vs f: OR = 0.96, 95% CI = 0.92–1.00), and mixed populations (Ff vs ff: OR = 1.25, 95% CI = 1.05–1.48; (FF + Ff) vs ff: OR = 1.15, 95% CI = 1.01–1.31) between the *VDR* FokI polymorphism and T2DM risk, as also shown in Table 2. However, after FPRP and BFDP correction, less-credible results were found in overall, Asians, African countries, Asian countries, and mixed populations, as also lists in Table 5.

**Table 5:** Credibility analysis of positive results in the present study.

				Credibility	
				Prior probability of 0.001	
Variables	Model	OR (95% CI)	*I*^2^ (%)	FPRP	BFDP
*VDR* BsmI polymorphism and T2DM risk					
Asian	BB vs (Bb + bb)	0.77 (0.60, 0.99)	40.5	0.979	0.998
VDR FokI polymorphism and T2DM risk					
Overall	FF vs ff	0.90 (0.84, 0.96)	51.6	0.579	0.990
	(FF + Ff) vs ff	0.98 (0.96, 1.00)	45.4	0.980	1.000
	FF vs (Ff + ff)	0.83 (0.75, 0.92)	62.4	0.280	0.954
	F vs f	0.93 (0.89, 0.97)	67.6	0.422	0.989
Asian	FF vs ff	0.87 (0.82, 0.93)	34.2	**0.041**	**0.797**
	(FF + Ff) vs ff	0.97 (0.94, 0.99)	0.0	0.775	0.999
	FF vs (Ff + ff)	0.79 (0.69, 0.90)	53.3	0.284	0.945
	F vs f	0.93 (0.90, 0.96)	45.9	**0.007**	**0.604**
Indian	(FF + Ff) vs ff	0.19 (0.07, 0.49)	–	0.992	0.981
	F vs f	0.28 (0.13, 0.62)	–	0.991	0.985
Africa	FF vs ff	0.77 (0.62, 0.96)	54.9	0.957	0.997
	FF vs (Ff + ff)	0.67 (0.56, 0.81)	51.1	**0.063**	**0.591**
Asia	FF vs ff	0.91 (0.84, 0.98)	50.5	0.927	0.999
	(FF + Ff) vs ff	0.97 (0.95, 1.00)	37.5	0.980	1.000
	FF vs (Ff + ff)	0.84 (0.75, 0.95)	56.9	0.846	0.995
	F vs f	0.94 (0.89, 0.99)	60.7	0.951	0.999
Sensitivity analysis for VDR FokI polymorphism and T2DM risk					
Overall	FF vs ff	0.93 (0.87, 1.00)	15.9	0.980	1.000
	FF vs (Ff + ff)	0.89 (0.81, 0.97)	21.8	0.888	0.997
	F vs f	0.96 (0.92, 1.00)	36.6	0.980	1.000
Asian	FF vs ff	0.92 (0.83, 1.00)	47.0	0.980	0.999
Caucasian	FF vs (Ff + ff)	0.85 (0.74, 0.98)	3.6	0.962	0.998
	F vs f	0.94 (0.88, 1.00)	37.3	0.980	1.000
Mixed	Ff vs ff	1.25 (1.05, 1.48)	–	0.907	0.996
	(FF + Ff) vs ff	1.15 (1.01, 1.31)	–	0.973	0.999
Africa	FF vs ff	0.85 (0.71, 1.00)	–	0.980	0.999
	FF vs (Ff + ff)	0.69 (0.53, 0.91)	–	0.935	0.994
	F vs f	0.84 (0.74, 0.95)	–	0.846	0.995
Asia	FF vs ff	0.92 (0.84, 1.00)	38	0.980	0.999
	F vs f	0.96 (0.92, 1.00)	32.7	0.980	1.000
VDR ApaI polymorphism and T2DM risk					
Caucasian	Aa vs aa	0.94 (0.89, 0.99)	0.0	0.951	0.999
	(Aa + AA) vs aa	0.96 (0.93, 1.00)	0.0	0.980	1.000
Mixed	AA vs (Aa + aa)	1.52 (1.04, 2.22)	–	0.985	0.997
North America	Aa vs aa	0.90 (0.81, 1.00)	55.3	0.980	0.999

BFDP = Bayesian false discovery probability, CI = confidence interval, FPRP = false-positive report probabilities, OR = odds ratio, T2DM = type 2 diabetes mellitus, VDR = vitamin D receptor. The positive results of VDR polymorphisms and type 2 diabetes mellitus risk.

### 3.4 Publication bias

**Figure 2:**
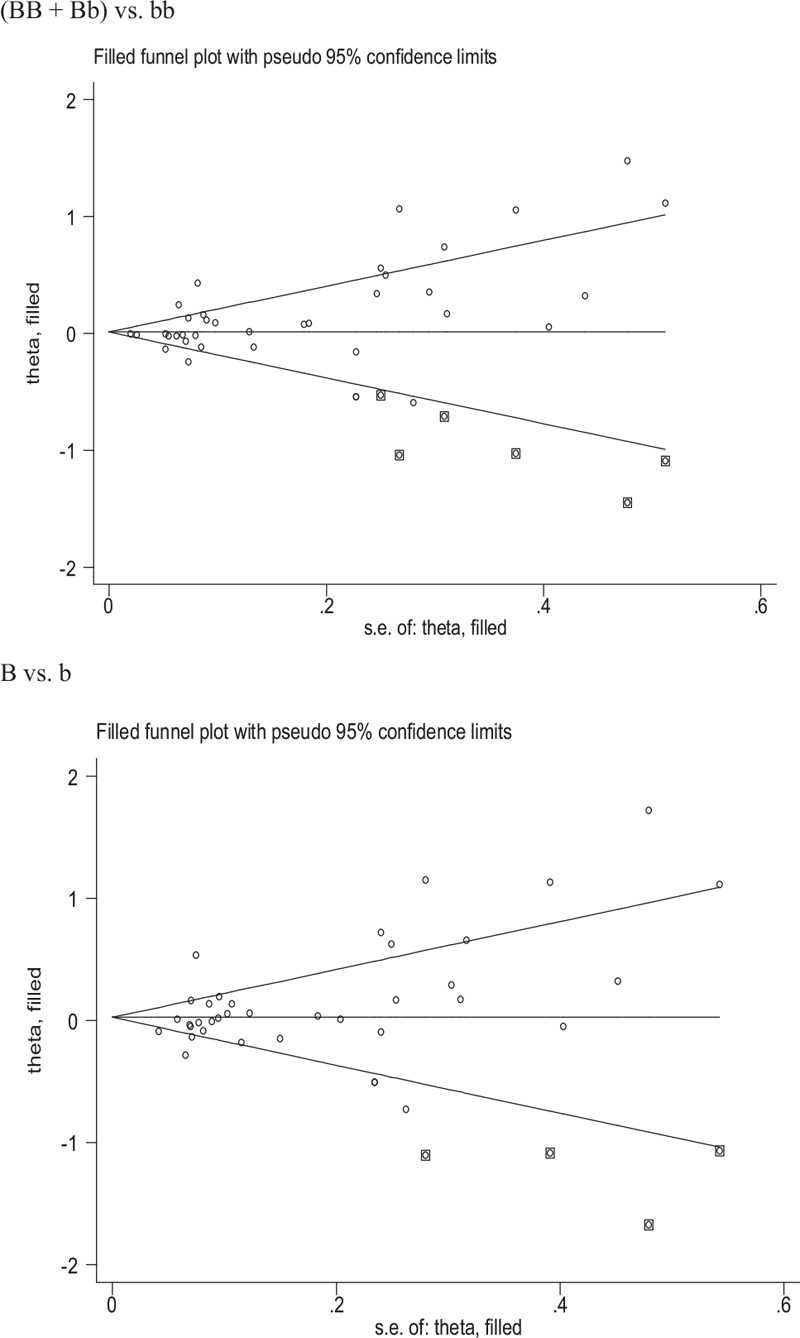
Begg funnel plot to assess publication bias.

Publication bias was only observed between the *VDR* BsmI polymorphism and T2DM risk by Begg funnel plot and Egger test ((BB + Bb) vs Bb: *P* = .041; B vs b: *P* = .044). Then, A nonparametric “trim and fill” method was used to adjust publication bias, We need to add 6 articles and 4 articles in the future for (BB + Bb) vs Bb and B vs b models, respectively, as shown in Figure 2. The results did not change for (BB + Bb) vs Bb and B vs b models (data not shown) in the overall analysis indicating that add studies cannot affect the merging results.

## 4 Discussion

T2DM is a chronic, complex, and life-long disease with a strong genetic component, which has a significant impact on quality of life, and increases the morbidity and mortality of other diseases, the etiology of T2DM is not elucidated till now. There were a lot of significant evidence indicating that the 4 *VDR* gene polymorphisms (BsmI, FokI, ApaI, and TaqI) have been considered as potential genetic factors for T2DM. However, the results from published studies are still inconsistent. Moreover, 4 previously published meta-analyses^[14–17]^ also have yielded obvious disagreement results, as lists in Table 3, Supplemental Digital Content. Thus, further evidence needs to clarify their associations with T2DM risk, and as far as we know that this is the first meta-analysis to explore the positive results by FPRP and BFDP test to avoid confounding factors.

Overall, the *VDR* BsmI polymorphism was associated with a significantly decreased T2DM risk in Asians; the *VDR* FokI polymorphism was associated with a significantly decreased T2DM risk in Asians, African countries, and Asian countries; the *VDR* ApaI polymorphism was associated with a significantly decreased T2DM risk in Caucasians and North American countries. On the *VDR* ApaI polymorphism, a significantly increased T2DM risk was found in a mixed population. The current study was performed by applying multiple subgroups and different genetic models, at the cost of multiple comparisons, in which case the pooled *P* value must be adjusted.^[81]^ FPRP was considered as an appropriate approach to evaluate the probability of significant results on the multiple hypothesis testing of gene polymorphism and disease susceptibility studies.^[24]^ In addition, Ioannidis JP et al^[25]^ provided a more precise Bayesian measure of false discovery in genetic epidemiology studies. Therefore, we employed FPRP and BFDP test to evaluate the false significant associations in this manuscript. Results of meta-regression analysis suggested that studies of ethnicity, sample size, quality score, and HWD were the source of heterogeneity. Deviation from HWE in controls may indicate selection bias, population stratification, or genotyping errors.^[82]^ In addition, random error and bias may be common in some small samples, low quality, and HWD in control studies, so that the results of these original researches can not be credible, especially in the studies of gene polymorphism and disease susceptibility. Moreover, as we know, small sample studies with significant results may be easier to accept than those with negative reports. However, when they tend to come positive results, their studies maybe not rigorous and often of low-quality. Hence, we assessed sensitivity analysis by including high-quality and HWE in control studies. However, when we further performed a sensitivity analysis, FPRP, and BFDP test, less-credible positive results were identified (all FPRP > 0.2 and BFDP > 0.8).

The *VDR* FokI polymorphism is located within the 5′ end of the gene near the promoter region. FokI polymorphism not only affects the function of the Vitamin D3 but also interrupts the binding efficiency of vitamin D and *VDR*, impairing insulin function and leading to T2DM finally. However, the single SNP role was much weak, this study indicates that significant association is less-credible positive results, we thought the *VDR* FokI polymorphism maybe not associated with T2DM risk. In addition. It has been indicated that the *VDR* TaqI polymorphism is a silent mutation despite being located in exon 9, and both BsmI and ApaI are located in the intron between exons 8 and 9 and do not alter the amount of the VDR protein, structure, or function.^[27]^ These biological functions supported our findings.

Table 3, Supplemental Digital Content shows the results of previously published meta-analyses on the association between the *VDR* (BsmI, FokI, ApaI, and TaqI) polymorphisms and T2DM risk. Yu et al^[14]^ in 2016 found that the *VDR* BsmI polymorphism significantly increased T2DM risk only in overall analysis; Zhu et al^[15]^ in 2014 and Li et al^[16]^ in 2013 reported that the *VDR* BsmI polymorphism was not associated with T2DM risk in overall populations, Asians and Caucasians; Wang et al^[17]^ in 2012 observed that the *VDR* BsmI polymorphism was associated with an increased T2DM risk in overall populations and Asians, as shown in Table 3, Supplemental Digital Content. Yu et al^[14]^ in 2016 reported that the FokI polymorphism significantly decreased T2DM risk in the overall analysis and Chinese population; Wang et al^[17]^ in 2012 observed that the *VDR* FokI polymorphism was associated with a decreased T2DM risk in overall populations and Asians; Li et al^[16]^ in 2013 found that the *VDR* FokI polymorphism was not consistently associated with either increased or decreased risk of T2DM in the overall analysis, as shown in Table 3, Supplemental Digital Content. Previously published meta-analyses did not found any significant association between the *VDR* (ApaI and TaqI) polymorphisms and T2DM risk, as shown in Table 3, Supplemental Digital Content. An obvious inconsistency was found in the classification of ethnic groups between these previously published meta-analyses and the present meta-analysis. Furthermore, all previously published meta-analyses did not adjusted ORs and their 95% CI. In addition, the sample size of this study was much larger. In the present study, 37 studies reported the *VDR* BsmI (5586 cases and 6484 controls), 31 studies examined the *VDR* FokI (6525 cases and 7464 controls), 19 studies investigated the *VDR* ApaI (2593 cases and 3557 controls), and 24 studies explored the *VDR* TaqI (3221 cases and 4027 controls) with T2DM risk. Previously meta-analyses reported the largest sample size only including 18 studies (2757 cases and 3517 controls) for the *VDR* BsmI, 12 studies (2218 cases and 1859 controls) for the *VDR* FokI, and 10 studies for the ApaI (1430 cases and 2441 controls), as shown also in Table 3, Supplemental Digital Content. In addition, Yu et al^[14]^ used 4 genetic models, Zhu et al^[15]^ applied 3 genetic models, and Li et al^[16]^ and Wang et al^[17]^ only employed 1 genetic model. Therefore, their results may be not credible.

The current meta-analysis, there has some advantages: (1) we assessed the quality of included studies; (2) we applied FPRP and BFDP test to evaluate the significant associations; (3) we explored sources of heterogeneity by meta-regression analysis; and (4) the sample size was larger over the previous meta-analysis. However, some potential limitations should be considered in the current meta-analysis. First, some potential covariates were not controlled, for example, age, gender, and so on. Second, in the subgroup analyses, the number of studies was small in Indians, North America, South America, and Africa, and there was not enough statistical power to explore their real associations. Third, T2DM is a complicated multi-genetic disease, the association was very weak between the single SNP and T2DM risk, unfortunately, no data were extracted on exploring the combined effects between gene and gene or gene and environment. Therefore, the study with a large sample size and a large enough subgroup will help to verify our findings.

In summary, this study strongly indicates that all significant associations were less credible positive results, rather than from true associations. Future larger-scale epidemiological investigations of this topic should be conducted to confirm or refute our findings.

## Author contributions

**Conceptualization:** Yao Liu, Hu-Wei Shen.

**Data curation:** Yao Liu, Xin Guo, Shao-Yan Huang.

**Formal analysis:** Yao Liu.

**Funding acquisition:** Yao Liu.

**Investigation:** Yao Liu.

**Methodology:** Yao Liu, Xiang-Hua Ye, Xiao-Feng He.

**Project administration:** Yao Liu, Xiao-Feng He.

**Resources:** Yao Liu.

**Software:** Yao Liu, Xiao-Feng He.

**Supervision:** Yao Liu, Luan Gong, Jin-Hui Cui, Hu-Wei Shen, Xiang-Hua Ye, Xiao-Feng He.

**Validation:** Yao Liu, Xiang-Hua Ye, Xiao-Feng He.

**Visualization:** Yao Liu.

**Writing – original draft:** Yao Liu, Hu-Wei Shen.

**Writing – review & editing:** Yao Liu, Hu-Wei Shen, Xiang-Hua Ye, Xiao-Feng He.

This study was designed by Xiao-Feng He, Hu-Wei Shen, and Xiang-Hua Ye. Yao Liu, Xin Guo, Shao-Yan Huang,Luan Gong, and Jin-Hui Cui did the literature search, study quality assessment, and data extraction. Yao Liu performed the statistical analysis, drafted the tables and figures, and wrote the first draft of this analysis, and Xiao-Feng He helped to finish the final version. All authors approved the conclusions of our study.

## Supplementary Material

Supplemental Digital Content
