# Supplementary material for: Evaluation of association studies and a systematic review and meta-analysis of VDR polymorphisms in type 2 diabetes mellitus risk
Source: Medicine (Baltimore). 2021 Jul 16;100(28):e25934. doi: 10.1097/MD.0000000000025934 (PMC8284732; doi:10.1097/MD.0000000000025934)
Supplement: Supplemental Digital Content [file medi-100-e25934-s001.doc]

Supplemental Table 1. Scale for quality assessment of molecular association studies of T2DM

| Criterion | Score |
| --- | --- |
| Source of case | |
| Selected from population | 2 |
| Selected from hospital | 1 |
| Not described | 0 |
| Source of control | |
| Population-based | 2 |
| Hospital-based | 1 |
| Not described | 0 |
| Ascertainment of T2DM | |
| Diagnosis of T2DM by WHO or ADA | 2 |
| Diagnosis of T2DM by patient medical record | 1 |
| Not described | 0 |
| Ascertainment of control | |
| Controls were tested to screen out T2DM | 2 |
| Controls were subjects who did not report T2DM, no objective testing | 1 |
| Not described | 0 |
| Matching | |
| Controls matched with cases by age and sex | 2 |
| Controls matched with cases only by age or sex | 1 |
| Not matched or not described | 0 |
| Genotyping examination | |
| Genotyping done blindly and quality control | 2 |
| Only genotyping done blindly or quality control | 1 |
| Unblinded and without quality control | 0 |
| HWE | |
| HWE in the control group | 2 |
| HWD in the control group | 0 |
| Association assessment | |
| Assess association between genotypes and T2DM with appropriate statistics and adjustment for confounders | 2 |
| Assess association between genotypes and T2DM with appropriate statistics without adjustment for confounders | 1 |
| Inappropriate statistics used | 0 |
| Total sample size |  |
| ≥ 200 | 2 |
| <200 | 0 |

HWE: Hardy-Weinberg equilibrium, HWD: Hardy-Weinberg disequilibrium, T2DM: type 2 diabetes mellitus

| **Supplemental Table 2. Included studies of VDR BsmI polymorphism in T2DM within the meta-analyses (A, Asian; I: Indian; Af, African; C, Caucasian; M, mixed; U, unidentified)** | | | | | | | | | | | | | | | | | |
| --- | --- | --- | --- | --- | --- | --- | --- | --- | --- | --- | --- | --- | --- | --- | --- | --- | --- |
| **No.** | **First Author/Year** | **Country** | **Eligible research studies of BsmI** | | | | | **Eligible research studies of FokI** | | | | **Eligible research studies of ApaI** | | | **Eligible research studies of TaqI** | | |
|  |  |  | **All studies** | | | | | **All studies** | | |  | **All studies** | | | **All studies** | | |
|  |  |  | **This study** | **Yu [16] 2016** | **Zhu [17] 2014** | **Li[18] 2013** | **Wang [19]2012** | **This study** | **Yu [16] 2016** | **Li[18] 2013** | **Wang [19]2012** | **This study** | **Li[18] 2013** | **Wang [19]2012** | **This study** | **Li[18] 2013** | **Wang [19]2012** |
| **1** | Boullu-Sanchis 1999 | India | – | – | – | – | – | – | – | – | – | I | A | Other | **I** | A | Other |
| **2** | Speer 2001 | Hungary | C | C | C | C | C | – | – | – | – | – | – | – | – | – | – |
| **3** | Ye WZ 2001 | France | C | C | C | C | C | – | – | – | – | C | C | C | C | C | C |
| **4** | Oh JY 2002 | USA | C(not in HWE) | C(not in HWE) | C(not in HWE) | C(not in HWE) | C(not in HWE) | – | – | – | – | C(not in HWE) | C(not in HWE) | C(not in HWE) | C(not in HWE) | C (not in HWE) | C(not in HWE) |
| **5** | Dong YH 2002 | China | – | – | – | – | – | – | – | – | – | A | – | A | A(not in HWE) | – | A(not in HWE) |
| **6** | Malecki MT 2003 | Poland | C | C | C | C | C | C | C | C | C | C | C | C | C | C | C |
| **7** | Shen BS 2004 | China | A | A | A | – | A | A | A | – | A | – | – | – | – | – | – |
| **8** | Li HM 2005 | China | – | – | – | – | – | A | A | – | A | – | – | – | – | – | – |
| **9** | Li HM 2005 | China | – | – | – | – | – | A | A | A | A | – | – | – | – | – | – |
| **10** | Liao L 2005 | China | **–** | – | – | – | – | A | A | – | A | – | – | – | – | – | – |
| **11** | Shi YJ 2007 | China | A | A | A | – | A | – | – | – | – | – | – | – | – | – | – |
| **12** | Xu JR 2007 | China | A | A | A | A | A | – | – | – | – | – | – | – | – | – | – |
| **13** | Zhai M 2008 | China | – | – | – | – | – | – | – | – | – | A | A | A | – | – | – |
| **14** | Du T 2008 | China | – | – | – | – | – | A | A | A | A | – | – | – | – | – | – |
| **15** | Zhang P 2008 | China | A | A | A | A | A | A | A | A | A | – | – | – | – | – | – |
| **16** | Bai R 2009 | China | – | – | – | – | – | A | A | – | A | – | – | – | – | – | – |
| **17** | Bid HK 2009 | India | I | C | I | A | Other | I(not in HWE) | C(not in HWE) | I(not in HWE) | Other(not in HWE) | – | – | – | I | I | Other |
| **18** | Wang CX 2009 | China | C | A | – | – | A | A | A | – | A | A | – | A | A | – | A |
| **19** | Ding HG 2009 | China | A | A | – | – | A | – | – | – | – | – | – | – | – | – | – |
| **20** | Lan XC 2009 | China | A | A | A | A | A | – | – | – | – | – | – | – | – | – | – |
| **21** | Nosratabadi R 2010 | Iran | – | – | – | – | – | – | – | – | – | C | – | Other | C(not in HWE) | – | Other(not in HWE) |
| **22** | Dilmec F 2010 | Turkish | – | – | – | – | – | – | – | – | – | C | C | C | C | C | C |
| **23** | Mukhopadhyaya PN 2010 | India | I | C | – | – | Other | – | – | – | – | – | – | – | I | – | Other |
| **24** | Su BC 2011 | China | A (not in HWE) | A (not in HWE) | – | – | – | A | A(not in HWE) | – | – | – | – | – | – | – | – |
| **25** | Nosratabadi R 2011 | Iran | – | – | – | – | – | – | – | – | – | C | C | – | C (not in HWE) | C (not in HWE) | – |
| **26** | Zhao Y 2011 | China | A | A | – | – | A | – | – | – | – | A | – | A | – | – | – |
| **27** | Al-Daghri NM 2012 | Saudi Arabia | C (not in HWE) | C (not in HWE) | – | – | – | C | C | – | – | – | – | – | – | – | – |
| **28** | Xu JR 2012 | China | A | A | – | – | – | – | – | – | – | – | – | – | – | – | – |
| **29** | Zhang H 2012 | China | A | A | – | – | – | – | – | – | – | – | – | – | – | – | – |
| **30** | Vedralova 2012 | Czech Republic | C | – | C | – | – | – | – | – | – | – | – | – | – | – | – |
| **31** | Polić MV 2012 | Croatia | M | – | – | – | – | – | – | – | – | Mixed | – | – | **Mixed** | – | – |
| **32** | XIA Zheng 2014 | China | A | – | – | – | – | A | – | – | – | – | – | – | – | – | – |
| **33** | J.R. Xu 2014 | China | A | – |  |  |  | – | – | – | – | – | – | – | **A** | – | – |
| **34** | Mackawy AM 2014 | Egypt | C (not in HWE) | – | – | – | – | C (not in HWE) | – | – | – | – | – | – | – | – | – |
| **35** | Al-Daghri NM 2014 | Saudi Arabia | C | – | – | – | – | C | – | – | – | C | – | – | **C** | – | – |
| **36** | Zhong X 2015 | China | A | – | – | – | – | A | – | – | – | A | – | – | – | – | – |
| **37** | Jia J 2015 | China | – | – | – | – | – | A | – | – | – | – | – | – | – | – | – |
| **38** | Rivera-Leon EA2015 | mexico | **–** | – | – | – | – | – | – | – | – | C(not in HWE) | – | – | **C** | – | – |
| **39** | Maia J 2016 | Brazil | **–** | – | – | – | – | C | – | – | – | – | – | – | **C** | – | – |
| **40** | Angel B 2016 | Chile | **–** | – | – | – | – | M | – | – | – | – | – | – | – | – | – |
| **41** | Mahjoubi I 2016 | Tunisia | **–** | – | – | – | – | C | – | – | – | – | – | – | – | – | – |
| **42** | Bertoccini L 2017 | Rome | **–** | – | – | – | – | C(not in HWE) | – | – | – | – | – | – | – | – | – |
| **43** | Rasheed MA 2017 | Egypt | C (not in HWE) | – | – | – | – | C | – | – | – | – | – | – | – | – | – |
| **44** | Yu F 2017 | China | A | – | – | – | – | A | – | – | – | – | – | – | – | – | – |
| **45** | Xia Z 2017 | China | – | – | – | – | – | A | – | – | – | A | – | – | **A** | – | – |
| **46** | Shab-Bidar S 2017 | Iran | C (not in HWE) | – | – | – | – | C(not in HWE) | – | – | – | C(not in HWE) | – | – | **C(not in HWE)** | – | – |
| **47** | Sarma D 2018 | India | I | – | – | – | – | I | – | – | – | – | – | – | **I** | – | – |
| **48** | Safar HA 2018 | United Arab Emirates | C | – | – | – | – | C | – | – | – | – | – | – | **C** | – | – |
| **49** | Malik R 2018 | India | I (not in HWE) | – | – | – | – | – | – | – | – | – | – | – | **I** | – | – |
| **50** | Angel B 2018 | Chile | M (not in HWE) | – | – | – | – | Mixed | – | – | – | – | – | – | – | – | – |
| **51** | Rodrigues KF 2019 | Brazil | C | – | – | – | – | C | – | – | – | C | – | – | **C** | – | – |
| **52** | Khan A 2019 | Pakistan | I (not in HWE) | – | – | – | – | – | – | – | – | – | – | – | – | – | – |
| **53** | Gendy HIE 2019 | Egypt | C | – | – | – | – | C | – | – | – | – | – | – | **C** | – | – |
| **54** | Fatma H 2019 | Pakistan | I (not in HWE) | – | – | – | – | – | – | – | – | – | – | – | – | – | – |
| **55** | Al-Hazmi AS 2019 | Saudi Arabia | C | – | – | – | – | – | – | – | – | C | – | – | **C** | – | – |
| **56** | Hatmal MM 2020 | Jordan | – | – | – | – | – | C | – | – | – | – | – | – | – |  | – |

| **Supplemental Table 3. Results of previous meta-analyses between the *VDR* polymorphisms and T2DM risk** | | | | | | | | | | | | |
| --- | --- | --- | --- | --- | --- | --- | --- | --- | --- | --- | --- | --- |
| First author/year | Variable | n (Cases/Controls) | BB vs. bb | | Bb vs. bb | | BB+Bb vs. bb | | BB vs. Bb+bb | | B vs. b | |
|  |  |  | OR (95% CI) | *P*_h_/*I*^2^ (%) | OR (95% CI) | *P*_h_/*I*^2^ (%) | OR (95% CI) | *P*_h_/*I*^2^ (%) | OR (95% CI) | *P*_h_/*I*^2^ (%) | OR (95% CI) | *P*_h_/*I*^2^ (%) |
|  |  |  |  |  |  |  |  |  |  |  |  |  |
| BsmI | | | | | | | | | | | | |
| Yu et al. [16] 2016 | Overall | 18 (2,757/3,517) | 1.01 (0.67, 1.52) | 0.002/57.9 | **1.36 (1.02, 1.83)** | 0/75.1 | **1.36 (1.00, 1.84)** | 0/79.6 | 0.93 (0.65, 1.33) | 0.002/57.3 | – | – |
|  | Chinese | 10 (1,413/2283) | 1.05 (0.30, 3.65) | – | 1.62 (0.97, 2.72) | – | 1.57 (0.88, 2.80) | – | 0.92 (0.32, 2.66) | – | – | – |
|  | Caucasian | 8 (1,344/1,234) | 1.13 (0.83, 1.54) | – | 1.15 (0.83, 1.59) | – | 1.17 (0.86, 1.89) | – | 1.02 (0.79, 1.32) | – | – | – |
| Zhu et al. [17] 2014 | Overall | 11 (1,650/2,608) | – | – | – | – | 1.33 (0.92, 1.96) | ＜0.001 | 0.87 (0.62–1.21) | 0.01 | 1.21 (0.89, 1.64) | ＜0.001 |
|  | Asian | 6 (–) | – | – | – | – | 1.69 (0.72, 4) | ＜0.001 | 1.07 (0.40–2.83) | 0.009 | 1.68 (0.73, 3.85) | ＜0.001 |
|  | Caucasian | 5 (–) | – | – | – | – | 1.02 (0.85, 1.23) | 0.41 | 0.96 (0.78–1.18) | 0.67 | 1.00 (0.88, 1.13) | 0.47 |
| Li et al. [18] 2013 | Overall | 8 (1,196/2,118) | – | – | – | – | – | – | – | – | 0.93 (0.66, 1.33) | 0/86.8 |
|  | Asian | 4 (–) | – | – | – | – | – | – | – | – | 0.65 (0.22, 1.97) | 0/94.2 |
|  | Caucasian | 4 (–) | – | – | – | – | – | – | – | – | 1.05 (0.92, 1.20) | 0.73/0 |
| Wang et al. [19] 2012 | Overall | 14 (1,778/2,800) | – | – | – | – | – | – | – | – | **1.41 (1.04, 1.92)** | ＜0.001/83.8 |
|  | Overall | 13 (–) | – | – | – | – | – | – | – | – | **1.49( 1.03, 2.15)** | ＜0.001/84.8 |
|  | Overall | 11 (–) | – | – | – | – | – | – | – | – | **1.52 (1.01, 2.28)** | ＜0.001/86.3 |
|  | Asian | 8 (–) | – | – | – | – | – | – | – | – | **2.60 (1.82, 3.72)** | 0.068/46.8 |
|  | Caucasian | 3 (–) | – | – | – | – | – | – | – | – | 0.90 (0.75, 1.07) | 0.684/0 |
|  | Other | 2 (–) | – | – | – | – | – | – | – | – | 0.55 (0.24, 1.25) | 0.030/78.8 |
| FokI | | | | | | | | | | | | |
| First author/year | Variable | n (Cases/Controls) | FF vs. ff | | FF vs. (Ff + ff) | | (FF + Ff) vs. ff | | F vs. f | | – | – |
|  |  |  | OR (95% CI) | *P*_h_/*I*^2^ (%) | OR (95% CI) | *P*_h_/*I*^2^ (%) | OR (95% CI) | *P*_h_/*I*^2^ (%) | OR (95% CI) | *P*_h_/*I*^2^ (%) | – | – |
|  |  |  |  |  |  |  |  |  |  |  | – | – |
| Yu et al. 2016[1] | Overall | 12 (2,218/1,859) | **0.64 (0.52, 0.78)** | 0.405/4.1 | **0.86 (0.74, 1.00)** | 0.177/27.3 | **0.64 (0.55, 0.74)** | 0.251/19.6 | – | – | – | – |
|  | Chinese | 9 (1,446/1,201) | **0.56 (0.44, 0.71)** | 0.619/0 | **0.79 (0.64, 0.97)** | 0.607/0 | **0.59 (0.50, 0.70)** | 0.330/12.6 | – | – | – | – |
|  | Caucasian | 3 (772/658) | 0.86 (0.59, 1.27) | 0.359/2.3 | 0.95(0.76, 1.19) | 0.024/73.1 | 0.83 (0.60, 1.12) | 0.531/0 | – | – | – | – |
| Li et al. 2013[3] | Overall | 5 (1,101/969) | **0.68 (0.52, 0.88)** | – | **0.66 (0.55, 0.80)** | – | 0.88 (0.69, 1.11) | – | **1.25 (1.10, 1.42)** | 0.257/24.6 | – | – |
|  | Asian | 4 (–) | – | – | – | – | – | – | **1.32 (1.14, 1.53)** | 0.37/9.3 | – | – |
|  | Caucasian | 1 (–) | – | – | – | – | – | – | 1.08 (0.85, 1.37) | – | – | – |
| Wang et al. 2012[4] | Overall | 10 (–) | – | – | – | – | – | – | **0.77 (0.69, 0.85)** | 0.173/29.6 | – | – |
|  | Overall | 9 (–) | – | – | – | – | – | – | **0.77 (0.69, 0.85)** | 0.124/36.8 | – | – |
|  | Asian | 8 (–) | – | – | – | – | – | – | **0.74 (0.65, 0.83)** | 0.203/28.2 | – | – |
|  | Caucasian | 1 (–) | – | – | – | – | – | – | 0.93 (0.73, 1.18) | – | – | – |
| ApaI | | | | | | | | | | | | |
| First author/year | Variable | n (Cases/Controls) | AA vs. aa | | AA vs. ( Aa +aa) | | (Aa + AA) vs. aa | | A vs. a | | – | – |
|  |  |  | OR (95% CI) | *P*_h_/*I*^2^ (%) | OR (95% CI) | *P*_h_/*I*^2^ (%) | OR (95% CI) | *P*_h_/*I*^2^ (%) | OR (95% CI) | *P*_h_/*I*^2^ (%) | – | – |
|  |  |  |  |  |  |  |  |  |  |  | – | – |
| Li et al. 2013[3] | Overall | 7 (1,206/2,175) | – | – | – | – | – | – | 1.02 (0.91, 1.13) | 0.186/31.7 | – | – |
|  | Asian | 3 (–) | – | – | – | – | – | – | 0.84 (0.66, 1.06) | 0/0 | – | – |
|  | Caucasian | 4 (–) | – | – | – | – | – | – | 1.07 (0.95, 1.22) | 0.157/42.4 | – | – |
| Wang et al. 2012[4] | Overall | 10 (1,430/2,441) | – | – | – | – | – | – | 0.97 (0.93, 1.02) | 0.279/13.8 | – | – |
|  | Overall | 9 (–) | – | – | – | – | – | – | 1 (0.88, 1.14) | 0.476/0 | – | – |
|  | Asian | 4 (–) | – | – | – | – | – | – | 0.99 (0.79, 1.25) | 0.269/23.7 | – | – |
|  | Caucasian | 3 (–) | – | – | – | – | – | – | 1.02 (0.86, 1.20) | 0.292/18.8 | – | – |
|  | Other | 2 (–) | – | – | – | – | – | – | 0.96 (0.72, 1.28) | 0.304/5.2 | – | – |
| TaqI | | | | | | | | | | | | |
| First author/year | Variable | n (Cases/Controls) | TT vs. tt | | TT vs. (Tt + tt) | | (Tt + TT) vs. tt | | T vs. t | | – | – |
|  |  |  | OR (95% CI) | *P*_h_/*I*^2^ (%) | OR (95% CI) | *P*_h_/*I*^2^ (%) | OR (95% CI) | *P*_h_/*I*^2^ (%) | OR (95% CI) | *P*_h_/*I*^2^ (%) | – | – |
|  |  |  |  |  |  |  |  |  |  |  | – | – |
| Li et al. 2013[3] | Overall | 7 (1220/2215) | – | – | – | – | – | – | 0.99(0.89, 1.11) | 0.367/8.1 | – | – |
|  | Asian | 3(–) | – | – | – | – | – | – | 1.16 (0.92, 1.45) | 0.350/4.7 | – | – |
|  | Caucasian | 4(–) | – | – | – | – | – | – | 0.95 (0.83, 1.08) | 0.518/0 | – | – |
| Wang et al. 2012[4] | Overall | 10 (1430, 2441) | – | – | – | – | – | – | 1.04 (0.88, 1.23) | 0.072/42.9 | – | – |
|  | Overall | 7(–) | – | – | – | – | – | – | 1.09 (0.85, 1.39) | 0.018/60.8 | – | – |
|  | Asian | 1(–) | – | – | – | – | – | – | 1.32 (0.59, 2.94) | – | – | – |
|  | Caucasian | 3(–) | – | – | – | – | – | – | 1.10 (0.93, 1.32) | 0.438/0 | – | – |
|  | other | 3(–) | – | – | – | – | – | – | 1.34 (0.57, 2.27) | 0.002/84.4 | – | – |

| Supplemental Table 4. Main characteristics and Quality score of studies included | | | | | | | | | | | | | |  |  |  |  |  |  |  |  |  |  |  |  |  |  |  |  |  |  |  |  |  |  |
| --- | --- | --- | --- | --- | --- | --- | --- | --- | --- | --- | --- | --- | --- | --- | --- | --- | --- | --- | --- | --- | --- | --- | --- | --- | --- | --- | --- | --- | --- | --- | --- | --- | --- | --- | --- |
| First Author/Year | Country | Geographic region | Ethnicity | Source of cases | Type of control | Matching | Quality score | Genotypes distribution BsmI | | | | | | HWE | Genotypes distribution FokI | | | | | | HWE | Genotypes distribution ApaI | | | | | | HWE | Genotypes distribution TaqI | | | | | | HWE |
|  |  |  |  |  |  |  |  | Cases | | | Controls | | |  | Cases | | | Controls | | |  | Cases | | | Controls | | |  | Cases | | | Controls | | |  |
|  |  |  |  |  |  |  |  | bb | Bb | BB | bb | Bb | BB |  | ff | Ff | FF | ff | Ff | FF |  | aa | Aa | AA | aa | Aa | AA |  | tt | Tt | TT | tt | Tt | TT |  |
| Boullu-Sanchis S 1999 | India | Asia | Indian | PB | Non-diabetic controls | Age and sex | 11 | – | – | – | – | – | – | – | – | – | – | – | – | – | – | 25 | 42 | 22 | 31 | 47 | 22 | 0.601 | 48 | 33 | 8 | 44 | 39 | 17 | 0.113 |
| Speer G 2001 | Hungary | Europe | Caucasian | HB | Healthy controls | No | 5 | 20 | 22 | 7 | 46 | 66 | 26 | 0.787 | – | – | – | – | – | – | – | – | – | – | – | – | – | – | – | – | – | – | – | – | – |
| Ye WZ 2001 | France | Europe | Caucasian | HB | Non-diabetic controls | No | 9 | 119 | 135 | 52 | 54 | 65 | 24 | 0.558 | – | – | – | – | – | – | – | 65 | 142 | 98 | 30 | 78 | 35 | 0.27 | 49 | 136 | 120 | 23 | 66 | 54 | 0.707 |
| Oh JY 2002 | USA | North America | Caucasian | PB | Non-diabetic controls | No | 9 | 86 | 107 | 49 | 460 | 590 | 253 | 0.01 | – | – | – | – | – | – | – | 66 | 92 | 84 | 264 | 552 | 487 | <0.001 | 41 | 108 | 93 | 219 | 581 | 503 | 0.021 |
| Dong YH 2002 | China | Asia | Asian | HB | Healthy controls | No | 10 | – | – | – | – | – | – | – | – | – | – | – | – | – | – | 37 | 25 | 6 | 33 | 24 | 5 | 0.829 | 63 | 5 | 0 | 58 | 3 | 1 | 0.003 |
| Malecki MT 2003 | Poland | Europe | Caucasian | PB | Non-diabetic controls | No | 11 | 131 | 142 | 35 | 92 | 116 | 32 | 0.63 | 64 | 159 | 85 | 52 | 110 | 77 | 0.284 | 84 | 153 | 71 | 56 | 124 | 60 | 0.603 | 30 | 140 | 138 | 31 | 117 | 92 | 0.512 |
| Shen BS 2004 | China | Asia | Asian | HB | Healthy controls | No | 8 | 59 | 34 | 3 | 45 | 7 | 0 | 0.603 | 19 | 53 | 24 | 10 | 24 | 18 | 0.694 | – | – | – | – | – | – | – | – | – | – | – | – | – | – |
| Li HM 2005 | China | Asia | Asian | HB | Healthy controls | No | 8 | – | – | – | – | – | – | – | 5 | 22 | 28 | 6 | 28 | 43 | 0.633 | – | – | – | – | – | – | – | – | – | – | – | – | – | – |
| Li HM 2005 | China | Asia | Asian | HB | Healthy controls | No | 7 | – | – | – | – | – | – | – | 19 | 46 | 39 | 6 | 28 | 43 | 0.633 | – | – | – | – | – | – | – | – | – | – | – | – | – | – |
| Liao L 2005 | China | Asia | Asian | PB | Healthy controls | No | 10 | – | – | – | – | – | – | – | 27 | 83 | 30 | 28 | 74 | 64 | 0.406 | – | – | – | – | – | – | – | – | – | – | – | – | – | – |
| Shi YJ 2007 | China | Asia | Asian | HB | Healthy controls | No | 8 | 139 | 17 | 1 | 177 | 18 | 1 | 0.47 | – | – | – | – | – | – | – | – | – | – | – | – | – | – | – | – | – | – | – | – | – |
| Xu JR 2007 | China | Asia | Asian | HB | Healthy controls | No | 9 | 19 | 46 | 41 | 6 | 28 | 68 | 0.192 | – | – | – | – | – | – | – | – | – | – | – | – | – | – | – | – | – | – | – | – | – |
| Zhai M 2008 | China | Asia | Asian | HB | Non-diabetic controls | Age and sex | 11 | – | – | – | – | – | – | – | – | – | – | – | – | – | – | 39 | 40 | 7 | 66 | 45 | 9 | 0.73 | – | – | – | – | – | – | – |
| Du T 2008 | China | Asia | Asian | HB | Healthy controls | No | 10 | – | – | – | – | – | – | – | 95 | 264 | 114 | 68 | 189 | 123 | 0.755 | – | – | – | – | – | – | – | – | – | – | – | – | – | – |
| Zhang P 2008 | China | Asia | Asian | PB | Healthy controls | Age and sex | 11 | 71 | 41 | 4 | 97 | 15 | 0 | 0.448 | 23 | 64 | 29 | 21 | 52 | 39 | 0.62 | – | – | – | – | – | – | – | – | – | – | – | – | – | – |
| Bai R 2009 | China | Asia | Asian | HB | Healthy controls | Age and sex | 9 | – | – | – | – | – | – | – | 22 | 50 | 34 | 7 | 26 | 44 | 0.286 | – | – | – | – | – | – | – | – | – | – | – | – | – | – |
| Bid HK 2009 | India | Asia | Indian | HB | Healthy controls | Age and sex | 12 | 30 | 52 | 18 | 60 | 77 | 23 | 0.831 | 38 | 60 | 2 | 80 | 79 | 1 | <0.001 | – | – | – | – | – | – | – | 15 | 49 | 36 | 28 | 65 | 67 | 0.085 |
| Wang CX 2009 | China | Asia | Asian | HB | Healthy controls | No | 8 | 56 | 8 | 0 | 110 | 11 | 0 | 0.6 | 15 | 29 | 20 | 19 | 65 | 37 | 0.278 | 36 | 21 | 7 | 55 | 46 | 20 | 0.061 | 1 | 7 | 56 | 0 | 22 | 99 | 0.271 |
| Ding HG 2009 | China | Asia | Asian | HB | Healthy controls | No | 4 | 19 | 13 | 0 | 26 | 4 | 0 | 0.696 | – | – | – | – | – | – | – | – | – | – | – | – | – | – | – | – | – | – | – | – | – |
| Lan XC 2009 | China | Asia | Asian | HB | Healthy controls | Age and sex | 9 | 48 | 13 | 5 | 75 | 5 | 0 | 0.773 | – | – | – | – | – | – | – | – | – | – | – | – | – | – | – | – | – | – | – | – | – |
| Nosratabadi R 2010 | Iran | Asia | Caucasian | PB | Healthy controls | Age and sex | 12 | – | – | – | – | – | – | – | – | – | – | – | – | – | – | 28 | 63 | 9 | 27 | 56 | 17 | 0.189 | 33 | 63 | 4 | 47 | 35 | 18 | 0.018 |
| Dilmec F 2010 | Turkish | Asia | Caucasian | HB | Healthy controls | Age and sex | 13 | – | – | – | – | – | – | – | – | – | – | – | – | – | – | 7 | 38 | 27 | 26 | 82 | 61 | 0.857 | 14 | 25 | 33 | 19 | 81 | 69 | 0.511 |
| Mukhopadhyaya PN 2010 | India | Asia | Indian | FB | Non-diabetic controls | No | 9 | 17 | 9 | 14 | 26 | 10 | 4 | 0.073 | – | – | – | – | – | – | – | – | – | – | – | – | – | – | 5 | 12 | 23 | 8 | 25 | 7 | 0.113 |
| Su BC 2011 | China | Asia | Asian | – | – | – | – | 264 | 21 | 3 | 118 | 15 | 6 | <0.001 | 34 | 221 | 33 | 16 | 95 | 28 | <0.001 | – | – | – | – | – | – | – | – | – | – | – | – | – | – |
| Nosratabadi R 2011 | Iran | Asia | Caucasian | PB | Healthy controls | Age and sex | 10 | – | – | – | – | – | – | – | – | – | – | – | – | – | – | – | – | – | – | – | – | – | 33 | 63 | 4 | 47 | 35 | 18 | 0.018 |
| Zhao Y 2011 | China | Asia | Asian | HB | Healthy controls | Age and sex | 10 | 67 | 29 | 0 | 71 | 11 | 1 | 0.455 | – | – | – | – | – | – | – | 28 | 63 | 9 | 27 | 56 | 17 | 0.189 | – | – | – | – | – | – | – |
| Al-Daghri NM 2012 | Saudi Arabia | Asia | Caucasian | PB | Healthy controls | No | 10 | 105 | 201 | 62 | 114 | 95 | 50 | <0.001 | 213 | 133 | 22 | 129 | 111 | 19 | 0.461 | – | – | – | – | – | – | – | – | – | – | – | – | – | – |
| Xu JR 2012 | China | Asia | Asian | PB | Healthy controls | No | 9 | 176 | 24 | 1 | 172 | 47 | 0 | 0.075 | – | – | – | – | – | – | – | – | – | – | – | – | – | – | – | – | – | – | – | – | – |
| Zhang H 2012 | China | Asia | Asian | HB | Healthy controls | No | 12 | 96 | 26 | 0 | 85 | 14 | 1 | 0.625 | – | – | – | – | – | – | – | 46 | 65 | 11 | 35 | 53 | 12 | 0.233 | – | – | – | – | – | – | – |
| Vedralova 2012 | Czech Republic | Europe | Caucasian | NR | Healthy controls | No | 5 | 14 | 47 | 43 | 20 | 33 | 30 | 0.079 | 18 | 60 | 38 | 12 | 76 | 25 | <0.001 | – | – | – | – | – | – | – | – | – | – | – | – | – | – |
| Polić MV 2012 | Croatia | Europe | Mixed | HB | Healthy controls | No | 9 | 8 | 12 | 5 | 15 | 15 | 10 | 0.132 | – | – | – | – | – | – | – | 3 | 14 | 8 | 5 | 25 | 11 | 0.115 | 5 | 10 | 10 | 8 | 15 | 17 | 0.184 |
| XIA Zheng 2014 | China | Asia | Asian | HB | Healthy controls | Age and sex | 12 | 120 | 14 | 0 | 82 | 8 | 1 | 0.143 | 8 | 60 | 66 | 9 | 47 | 35 | 0.234 | – | – | – | – | – | – | – | – | – | – | – | – | – | – |
| J.R. Xu 2014 | China | Asia | Asian | HB | Non-diabetic controls | No | 12 | 122 | 30 | 2 | 87 | 28 | 0 | 0.137 | – | – | – | – | – | – | – | – | – | – | – | – | – | – | 3 | 17 | 134 | 0 | 16 | 99 | 0.423 |
| Mackawy AM 2014 | Egypt | Africa | Caucasian | HB | Healthy controls | Age and sex | 8 | 8 | 17 | 42 | 6 | 14 | 40 | 0.016 | 23 | 17 | 27 | 5 | 11 | 44 | 0.005 | – | – | – | – | – | – | – | – | – | – | – | – | – | – |
| Al-Daghri NM 2014 | Saudi Arabia | Asia | Caucasian | NR | Healthy controls | No | 10 | – | – | – | – | – | – | – | – | – | – | – | – | – | – | – | – | – | – | – | – | – | – | – | – | – | – | – | – |
| Zhong X 2015 | China | Asia | Asian | HB | Healthy controls | Age and sex | 11 | 77 | 27 | 6 | 96 | 18 | 2 | 0.301 | 35 | 61 | 14 | 40 | 58 | 18 | 0.688 | 16 | 60 | 34 | 28 | 59 | 29 | 0.852 | – | – | – | – | – | – | – |
| Jia J 2015 | China | Asia | Asian | PB | Non-diabetic controls | Age and sex | 14 | – | – | – | – | – | – | – | 212 | 336 | 120 | 579 | 973 | 408 | 0.983 | – | – | – | – | – | – | – | – | – | – | – | – | – | – |
| Rivera-Leon EA 2015 | mexico | North America | Mixed | PB | Healthy controls | No | 10 | – | – | – | – | – | – | – | – | – | – | – | – | – | – | 14 | 64 | 47 | 16 | 78 | 31 | 0.003 | 25 | 62 | 38 | 19 | 72 | 34 | 0.059 |
| Maia J 2016 | Brazil | South America | Caucasian | PB | Non-diabetic controls | Age and sex | 11 | – | – | – | – | – | – | – | – | – | – | – | – | – | – | – | – | – | – | – | – | – | 6 | 39 | 37 | 12 | 39 | 49 | 0.336 |
| Angel B 2016 | Chile | South America | Mixed | HB | NR | Sex | 10 | – | – | – | – | – | – | – | 40 | 96 | 24 | 32 | 75 | 53 | 0.56 | – | – | – | – | – | – | – | – | – | – | – | – | – | – |
| Mahjoubi I 2016 | Tunisia | Africa | Caucasian | HB | Non-diabetic controls | No | 10 | – | – | – | – | – | – | – | 231 | 180 | 28 | 168 | 117 | 17 | 0.565 | – | – | – | – | – | – | – | – | – | – | – | – | – | – |
| Bertoccini L 2017 | Rome | Europe | Caucasian | HB | Non-diabetic controls | No | 9 | – | – | – | – | – | – | – | 395 | 379 | 109 | 378 | 359 | 93 | 0.004 | – | – | – | – | – | – | – | – | – | – | – | – | – | – |
| Rasheed MA 2017 | Egypt | Africa | Caucasian | HB | Healthy controls | Age and sex | 12 | 28 | 36 | 23 | 57 | 52 | 41 | <0.001 | 12 | 38 | 37 | 11 | 47 | 92 | 0.158 | – | – | – | – | – | – | – | – | – | – | – | – | – | – |
| Yu F 2017 | China | Asia | Asian | PB | Non-diabetic controls | Age and sex | 15 | 354 | 43 | 0 | 698 | 75 | 3 | 0.52 | 80 | 205 | 112 | 147 | 405 | 223 | 0.124 | – | – | – | – | – | – | – | – | – | – | – | – | – | – |
| Xia Z 2017 | China | Asia | Asian | PB | Healthy controls | Age and sex | 14 | – | – | – | – | – | – | – | 66 | 60 | 8 | 38 | 50 | 12 | 0.468 | 75 | 50 | 9 | 49 | 38 | 13 | 0.205 | 127 | 7 | 0 | 86 | 14 | 0 | 0.452 |
| Shab-Bidar S 2017 | Iran | Asia | Caucasian | HB | Healthy controls | No | 8 | 41 | 211 | 106 | 37 | 189 | 146 | 0.031 | 71 | 118 | 169 | 62 | 119 | 191 | <0.001 | 56 | 166 | 126 | 43 | 210 | 119 | <0.001 | 71 | 160 | 127 | 75 | 139 | 158 | <0.001 |
| Sarma D 2018 | India | Asia | Asian | HB | Healthy controls | Age and sex | 10 | 12 | 23 | 5 | 10 | 6 | 4 | 0.128 | 32 | 0 | 8 | 15 | 3 | 2 | 0.032 | – | – | – | – | – | – | – | 22 | 10 | 8 | 14 | 4 | 2 | 0.094 |
| Safar HA 2018 | United Arab Emirates | Asia | Caucasian | HB | Healthy controls | No | 10 | 67 | 118 | 78 | 33 | 38 | 20 | 0.16 | 20 | 94 | 147 | 18 | 34 | 38 | 0.052 | – | – | – | – | – | – | – | 108 | 111 | 43 | 37 | 38 | 16 | 0.261 |
| Malik R 2018 | India | Asia | Indian | HB | Healthy controls | No | 10 | 5 | 16 | 79 | 38 | 22 | 40 | <0.001 | – | – | – | – | – | – | – | – | – | – | – | – | – | – | 19 | 45 | 37 | 15 | 39 | 46 | 0.171 |
| Angel B 2018 | Chile | South America | Mixed | PB | Non-diabetic controls | Age | 14 | 77 | 36 | 25 | 97 | 49 | 26 | <0.001 | 28 | 86 | 24 | 53 | 81 | 38 | 0.504 | – | – | – | – | – | – | – | – | – | – | – | – | – | – |
| Rodrigues KF 2019 | Brazil | South America | Caucasian | HB | Non-diabetic controls | Age and sex | 12 | 16 | 49 | 36 | 9 | 33 | 20 | 0.435 | 61 | 31 | 9 | 31 | 24 | 7 | 0.482 | 60 | 33 | 8 | 38 | 22 | 2 | 0.578 | 10 | 47 | 44 | 9 | 32 | 21 | 0.569 |
| Khan A 2019 | Pakistan | South America | Caucasian | HB | Non-diabetic controls | Age and sex | 12 | 116 | 69 | 65 | 79 | 139 | 32 | 0.016 | – | – | – | – | – | – | – | – | – | – | – | – | – | – | – | – | – | – | – | – | – |
| Gendy HIE 2019 | Egypt | Asia | Indian | HB | Healthy controls | No | 7 | 10 | 25 | 15 | 5 | 24 | 21 | 0.623 | 8 | 26 | 16 | 3 | 15 | 32 | 0.498 | – | – | – | – | – | – | – | 7 | 24 | 19 | 7 | 20 | 23 | 0.442 |
| Fatma H 2019 | Pakistan | Africa | Caucasian | HB | Healthy controls | Age | 9 | 4 | 119 | 27 | 2 | 64 | 34 | <0.001 | – | – | – | – | – | – | – | – | – | – | – | – | – | – | – | – | – | – | – | – | – |
| Al-Hazmi AS 2019 | Saudi Arabia | Asia | Indian | HB | Non-diabetic controls | No | 6 | 14 | 41 | 45 | 12 | 38 | 50 | 0.264 | – | – | – | – | – | – | – | 21 | 47 | 32 | 22 | 48 | 30 | 0.735 | 7 | 24 | 19 | 7 | 20 | 23 | 0.442 |
| Hatmal MM 2020 | Jordan | Asia | Caucasian | PB | Healthy controls | No | 11 | – | – | – | – | – | – | – | 7 | 31 | 44 | 6 | 27 | 49 | 0.406 | – | – | – | – | – | – | – | – | – | – | – | – | – | – |
| HB hospital-based study, PB population-based study, FB family-based study, NR not reported | | | | | | | | | | | | | | | | | | | | | | | | | | | | | | | | | | | |
